# Subcortical language localization using sign language and awake craniotomy for dominant posterior temporal glioma resection in a hearing-impaired patient

**DOI:** 10.1007/s00701-023-05586-7

**Published:** 2023-04-20

**Authors:** Ruth Lau, Armaan K Malhotra, Mary Pat McAndrews, Paul Kongkham

**Affiliations:** 1 Division of Neurosurgery, Krembil Brain Institute, Toronto Western Hospital, University Health Network, Toronto, Canada; 2 Department of Psychology, Krembil Research Institute, University Health Network, Toronto, Canada

**Keywords:** Intraoperative direct cortical stimulation, Awake mapping, Hearing-impaired patient, Sign language

## Abstract

Intraoperative direct cortical stimulation (DCS) is the gold standard technique to maximize the extent of resection of tumors located in eloquent areas. To date, there are three cases reported of awake mapping for language centers in deaf patients who could communicate only with sign language. We present the case of DCS in a deaf patient who could communicate vocally, native to American Sign Language and English, that underwent intraoperative awake mapping. DCS showed similar disruption of expressive phonology to both pictorial and gestural stimuli, confirming that sign language follows the same pattern as oral language.

## Introduction

In patients with intrinsic brain tumors involving eloquent areas, intraoperative direct cortical stimulation (DCS) is considered the gold standard technique to maximize the extent of resection while preserving function when feasible. One such application of intraoperative DCS is to maximize resection in lesions associated with critical language regions. However, few cases have been reported using awake mapping techniques for language centers in deaf patients [6]. To our knowledge, there are two cases reported of DCS in a native prelingual deaf-mute signer [13] and one case in a post-lingual deaf-mute signer [3, 11]. Sign language was tested intraoperatively in these cases, and none of these patients could communicate vocally.

We present the case of awake DCS in a post-lingual deaf patient who was able to communicate vocally, native to American Sign Language and English, who underwent intraoperative awake mapping for both languages. This is the first case report where both modalities, sign language and spoken language, were tested in the same patient during awake mapping. Furthermore, we describe a pre-operative functional MRI (fMRI) and baseline naming approach for language mapping and discuss the neuroscientific implications of our awake mapping findings for hearing-impaired patients.

## Case report

We report the case of a 66-year-old right-handed gentleman with no hearing on the right side and profound sensorineural hearing loss on the left side since childhood; he had some serviceable hearing function on the left side using a hearing aid, was a fluent communicator with American Sign Language (ASL) and had functional comprehensible speech in English. He was also proficient in lip-reading. The patient provided informed consent to participate in this case report.

Following initial presentation with a focal impaired awareness seizure, magnetic resonance imaging (MRI) revealed an intra-axial lesion involving the posterior aspect of the left temporal lobe extending to the angular gyrus with local gyrus expansion, blurring of the grey-white junction and mass effect causing effacement of the surrounding sulci. The lesion did not demonstrate gadolinium enhancement and was hypointense on T1-weighted sequences and hyperintense on T2-weighted imaging and FLAIR. The lesion had characteristics consistent with our working diagnosis of lower-grade glioma. However, given the patient’s age, there was a concern for an increased risk of this lesion being higher grade in nature.

**Fig. 1:**
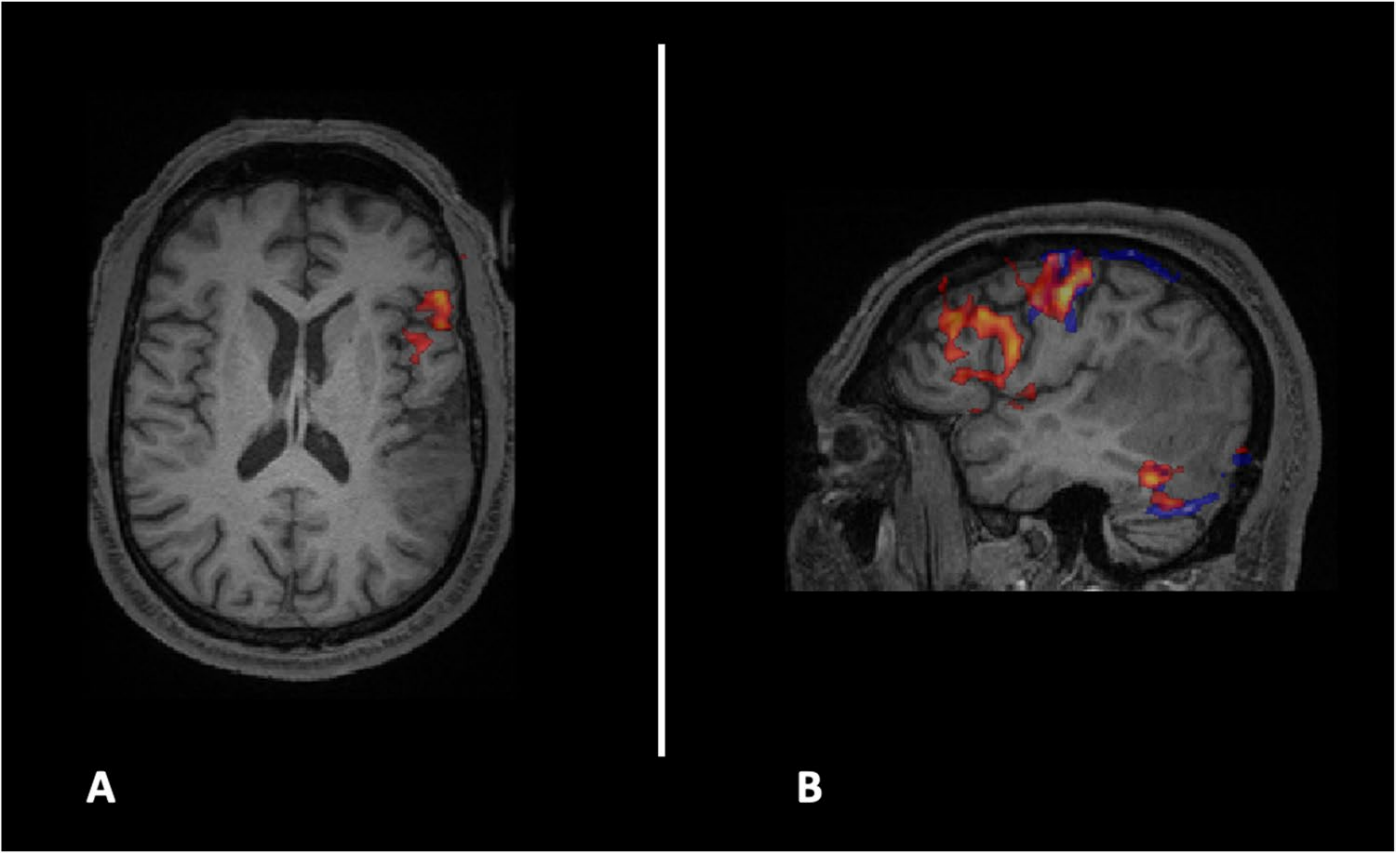
Functional magnetic resonance imaging showing (**A**, axial view; **B**: sagittal view) lateralization of the language to the left hemisphere, with activations near the lesion in the left temporal posterior region. Red corresponds to the sentence completion task; blue corresponds to the antonym generation task

Preoperative studies were completed, including task-based functional MRI (fMRI) and diffusion tensor imaging (DTI) for tractography reconstruction, as well as a baseline assessment of naming for stimuli to be used during DCS. In addition, two visual tasks were used to assess language in fMRI, sentence completion and antonym generation; both required covert responses. As shown in Fig. 1, language was lateralized to the left hemisphere, with some activations in proximity to the lesion.

The tractography reconstruction was performed with the Modus Plan™ software (Synaptive Medical®, Canada), using a fractional anisotropy set to an arbitrary threshold of 0.15 [2].

**Fig. 2:**
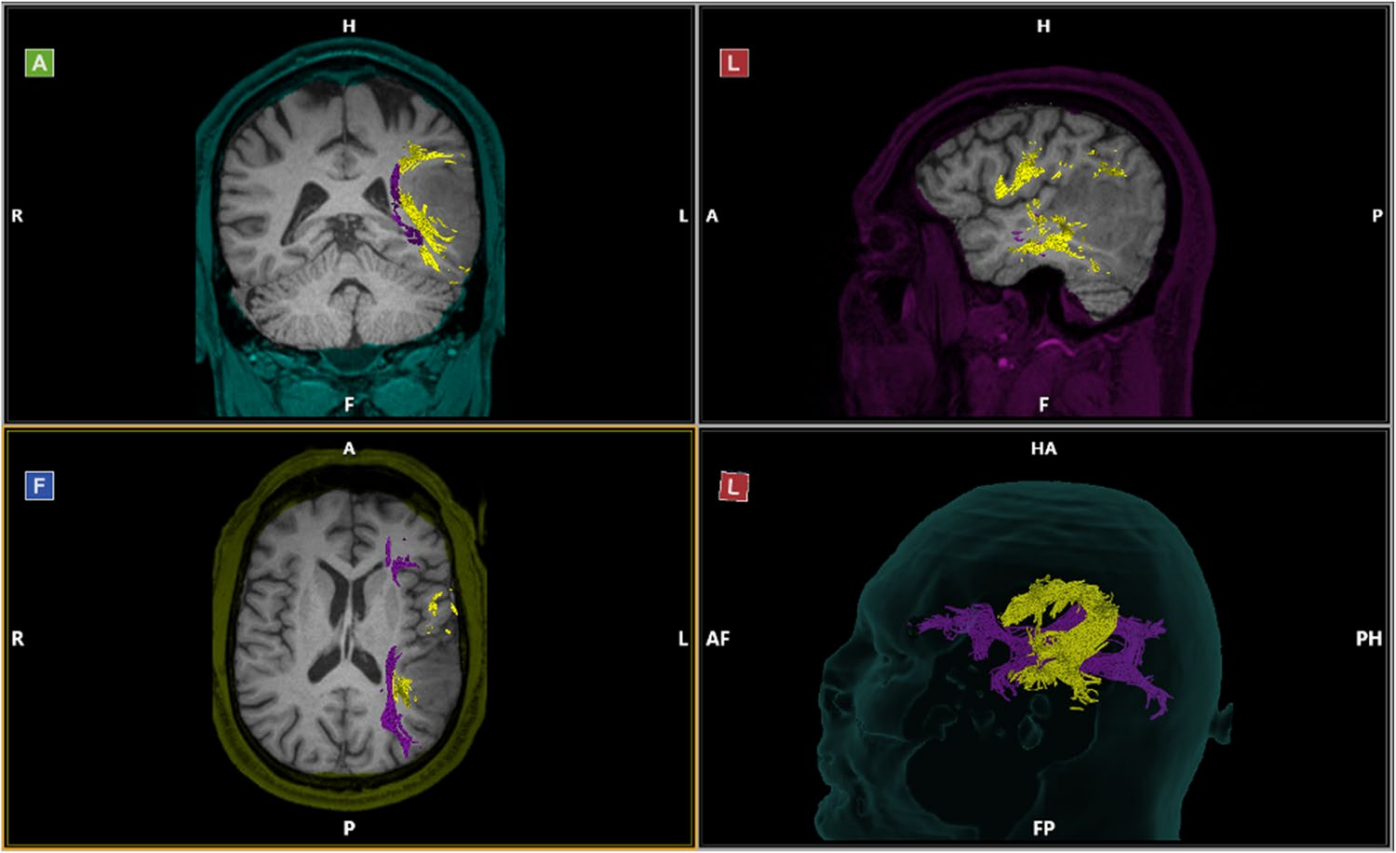
T1-weighted sequence showing a left temporal posterior non-enhancing lesion. The AF (yellow) is shown, surrounding the lesion anteriorly and medially. The IFOF (purple) is intimately related to the lesion’s inferior and medial borders

The DTI revealed a white matter tract consistent with the inferior fronto-occipital fasciculus (IFOF) located inferior and medial to the lesion, as well as arcuate fasciculus (AF) fibers anterior and medial to the lesion (Fig. 2). Given the proximity of these critical language pathways and the patient’s candidacy for awake language mapping, we elected to proceed with awake craniotomy for resection and had plans to test verbal and sign language modalities.

Under conscious sedation, the patient was positioned supine, his head fixed in a Sugita frame, and his head turned 30° to the right side. Local anesthetic was administered at the pin sites, as well as a local operative field block circumferentially around our planned operative site based on neuronavigation. Neuromonitoring to facilitate intraoperative cortical/subcortical language mapping was established. An awake anesthetic with conscious sedation was administered using dexmedetomidine. Sterile draping of the surgical field was performed using a mayo stand to lift the drapes away from the patient’s face. The right arm and hand were freely movable, and an iPad with visual stimuli was visible to the patient. Once the craniotomy had been performed, the patient was awakened for language mapping. We performed cortical mapping using an Ojemann bipolar handheld probe with 5-mm spacing between the probe tips, delivering biphasic current (square-wave pulses in 4-s trains at 60 Hz, single-pulse phase duration 1 ms, and amplitude 1–10 mA) to the brain.

Language mapping was performed using a testing platform, Neuromapper, developed by Dr. Sabsevitz. Stimulation was followed 500 ms later by the presentation of a new visual stimulus with the prompt for the patient to name the presented object verbally. Naming with sign language, which would require bimanual dexterity, was not tested, given the constraints of the surgical procedure setting. Instead, vocal naming was tested for both languages, with both pictures and ASL gestures as stimuli, through collaboration intraoperatively with neuropsychology and an ASL translator.

**Fig. 3:**
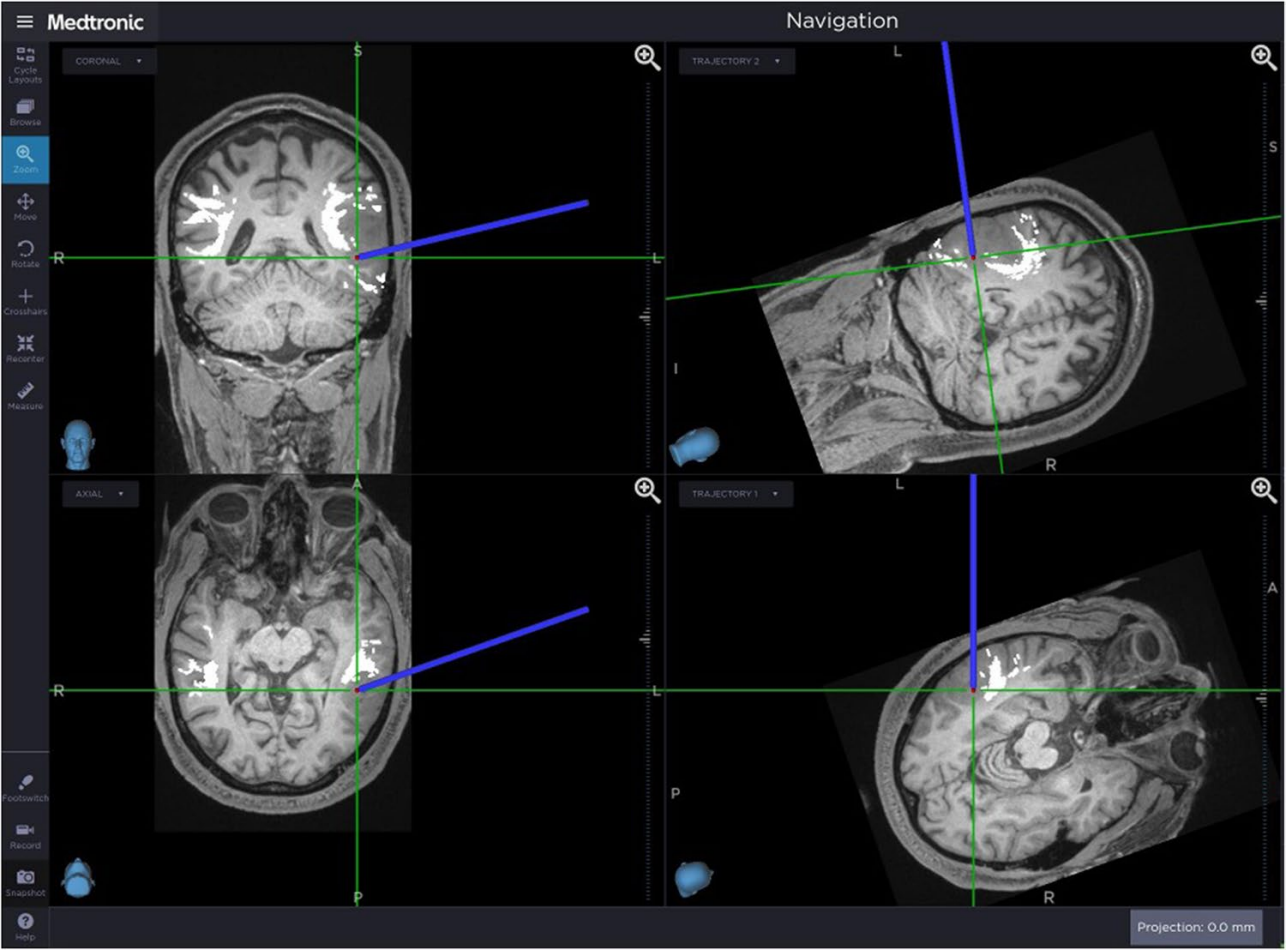
Neuro-navigation demonstrates the probe location at the level of the medial wall of the resection, where phonemic paraphasias were elicited with subcortical stimulation. With the overlaid DTI maps, there is evident proximity to the AF and IFOF

**Fig. 4:**
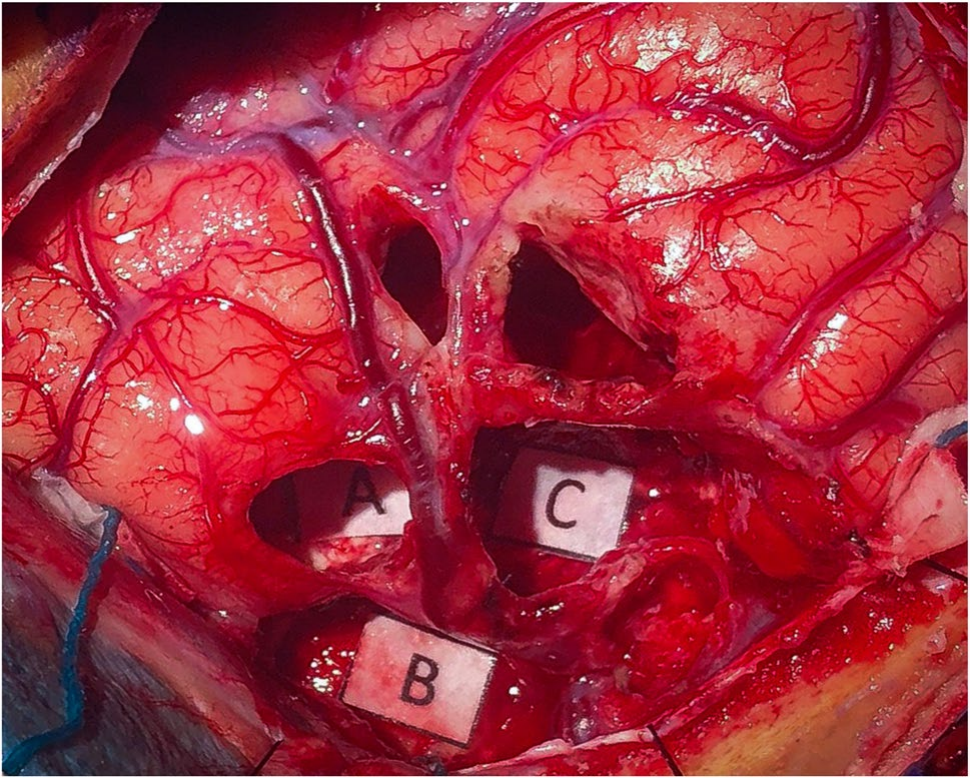
Intra-operative image of the resection cavity. **A**, **B**, and **C** correspond to the points where phonemic paraphasias were elicited

The result of the cortical mapping was negative across both modalities. Therefore, the resection was started, and subcortical mapping was serially performed at various stages of tumor removal. While advancing the resection, we noticed that the subcortical mapping at the medial margin of the tumor elicited phonemic paraphasias, including phonemic substitutions, neologisms, and perseverations of initial phonemes for both types of cues (pictures and ASL gestures). The gross anatomical location and neuro-navigation (which included pre-operative DTI maps) confirmed that these paraphasias occurred during stimulation at the level of the AF and IFOF (Figs. 3 and 4). As a result, this intra-operative location marked the medial extent of our resection.

**Fig. 5:**
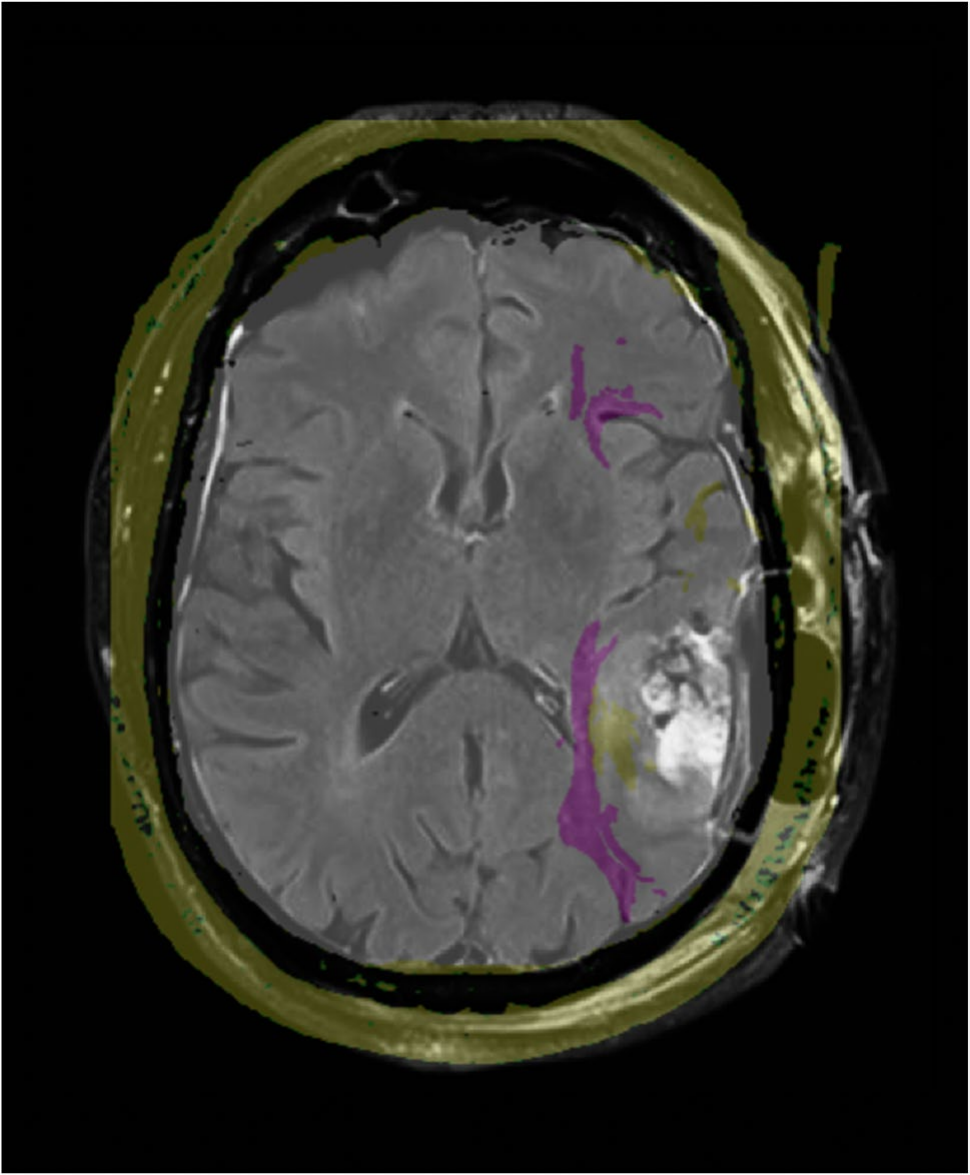
FLAIR sequence of the postoperative magnetic resonance imaging showing gross total resection of the tumor. The preoperative DTI has been superimposed, indicating that the medial resection was stopped at the level of the AF (yellow) and the IFOF (purple)

The postoperative MRI demonstrated gross total resection (Fig. 5). The histopathological report confirmed the diagnosis of glioblastoma IDH-wildtype, WHO grade 4. Postoperatively, the patient presented with transient neurologic deficits of both phonemic and semantic paraphasias in sign and spoken languages, which had resolved mainly by the second postoperative day.

## Discussion

Hearing loss can be either congenital or, more commonly, acquired after birth and is described in relation to spoken language acquisition as pre-lingual or post-lingual [5]. Many individuals use sign language to overcome speech and language difficulties and utilize both hands, body movements, and facial expressions. Results from studies in deaf patients have demonstrated functional reorganization aligning with the concept of cross-modal plasticity, whereby cortical re-organization occurs following the loss of sensory modality input [1, 15, 16]. Enhancement of non-auditory skills has been associated with compensation from other brain regions [1] involving a robust ventral pathway with improved emotional facial recognition and peripheral visual field tasks [12, 14]. In addition, the use of sign language itself has been associated with increased grey matter volume at the area of the hand knob in the precentral gyrus [7–9].

A recent study found that early language acquisition, whether oral or signed, enabled the normal development of the different white matter tracts involved in language [4]. In contrast, early language deprivation was found to be related to alterations in the left dorsal AF pathway [4]. The findings in our patient, who had subcortical stimulation and tractography consistent with normal anatomy of the AF and who had early access to language, are compatible with the literature, supporting the early relationship between language access and language network reorganization.

The feasibility of performing awake DCS with sign language for glioma resection in deaf-mute signers has been previously reported [3, 11, 13] and recommended [6] in the literature. In these cases, patients were able to communicate only with sign language and demonstrated a similar distribution between speech production and speech processing pathways in deaf and non-deaf patients[11, 13].

Our case is the first to report a deaf patient native to sign language and English, able to communicate vocally, where verbal responses to both pictures and sign gestures were tested using intraoperative DCS. Furthermore, our pre-operative structural and functional imaging correlated with intra-operative subcortical stimulation, emphasizing the importance of these pre-operative investigations. We elicited classic paraphasias, including neologisms, by stimulating the medial portion of the resection cavity. Furthermore, our intra-operative navigation corresponded anatomically to the AF and the IFOF. We corroborated that the sign language followed the same pattern as the spoken language, as we identified the same type of error in the same areas for both types of expression. This suggests a similar phonological organization in deaf patients fluent in sign language [6, 10, 11, 13]. In addition, the postoperative edema adjacent to the cavity resection, involving the IFOF and the AF, contributed to similar transient paraphasias for both ASL and spoken language following surgery, which recapitulates the proximity of spoken and gestural language centers.

## Conclusions

This case shows the feasibility of awake surgery with cortical and subcortical mapping, testing receptive and expressive speech in hearing-impaired patients who communicate with sign and oral language. Furthermore, neuroimaging and DCS for language mapping in awake craniotomies of deaf signers with brain tumors suggest that sign language follows the same localization as spoken language. Our report contributes to this body of knowledge and, importantly, demonstrates the similar disruption of expressive phonology to both pictorial and gestural stimuli. Therefore, we recommend treating brain lesions in the dominant hemisphere language regions of deaf patients as if they were vocal language speakers and emphasizing the correlation between pre-operative language and imaging studies with operative strategies.
